# Time to reconsider the role of ribavirin in Lassa fever

**DOI:** 10.1371/journal.pntd.0009522

**Published:** 2021-07-08

**Authors:** Alex Paddy Salam, Vincent Cheng, Tansy Edwards, Piero Olliaro, Jonathan Sterne, Peter Horby

**Affiliations:** 1 Centre for Tropical Medicine and Global Health, Nuffield Department of Medicine, University of Oxford, Oxford, United Kingdom; 2 United Kingdom Public Health Rapid Support Team, London, United Kingdom; 3 Population Health Sciences, Bristol Medical School, University of Bristol, Bristol, United Kingdom; 4 London School of Hygiene and Tropical Medicine, London, United Kingdom; Aix-Marseille Universite, FRANCE

## Abstract

Ribavirin is the only available Lassa fever treatment. The rationale for using ribavirin is based on one clinical study conducted in the early 1980s. However, reanalysis of previous unpublished data reveals that ribavirin may actually be harmful in some Lassa fever patients. An urgent reevaluation of ribavirin is therefore needed.

Fifty years after its discovery, Lassa fever remains uncontrolled, and mortality remains unacceptably high. Since 2015, Nigeria has been experiencing increasingly large outbreaks of Lassa fever, with new peaks reached in 2016, 2017, and 2018. In 1987, McCormick and colleagues reported a case fatality rate (CFR) of 16.5% among 441 patients hospitalized in Sierra Leone [1]. In Nigeria in 2019, 124 deaths were recorded among 554 laboratory-confirmed cases for a CFR of 22% [2].

Ribavirin is the only available Lassa fever–specific treatment and has been used routinely for over 25 years. However, intravenous ribavirin is not licensed for Lassa fever. Its mechanism of action is unclear, it is expensive and hard to source, and it has well-known toxicities [3]. Therefore, the evidence for using ribavirin in Lassa fever deserves careful scrutiny. The emergence of potential new therapeutics for Lassa fever, such as favipiravir and monoclonal antibodies, adds further weight to the case for reconsidering the role of ribavirin since the evaluation of new drugs in clinical trials requires a comparison against existing treatments with a known efficacy and safety profile [4,5].

The rationale for using ribavirin in Lassa fever is primarily based on one clinical study conducted in Sierra Leone in the late 1970s and early 1980s. McCormick and colleagues [6] reported that in Lassa fever patients with a serum aspartate aminotransferase (AST) level of ≥150 IU/L, the use of intravenous ribavirin within the first 6 days of illness reduced the fatality rate from 61% (11/18) with no ribavirin to 5% (1/20) (*p* = 0.002). These authors concluded that ribavirin is effective in the treatment of Lassa fever. However, there are long-standing concerns about the methods used in this study. Although randomization was used to assign patients to treatment groups, the comparisons presented were not according to original randomized groups, and we have reconstructed their derivation (Fig 1). Serious limitations to the comparisons presented include the use of historic controls, inclusion of pregnant women in the control group but their exclusion from the ribavirin group (case fatality is around 2-fold higher in pregnant women than nonpregnant patients), and post hoc merging of treatment groups. Despite this and the fact that the results only supported the use of ribavirin in nonpregnant adult patients with AST ≥150 IU/L, this study is the basis upon which ribavirin is now used in all patients with Lassa fever, including children, pregnant women, and people with normal liver function.

**Fig 1:**
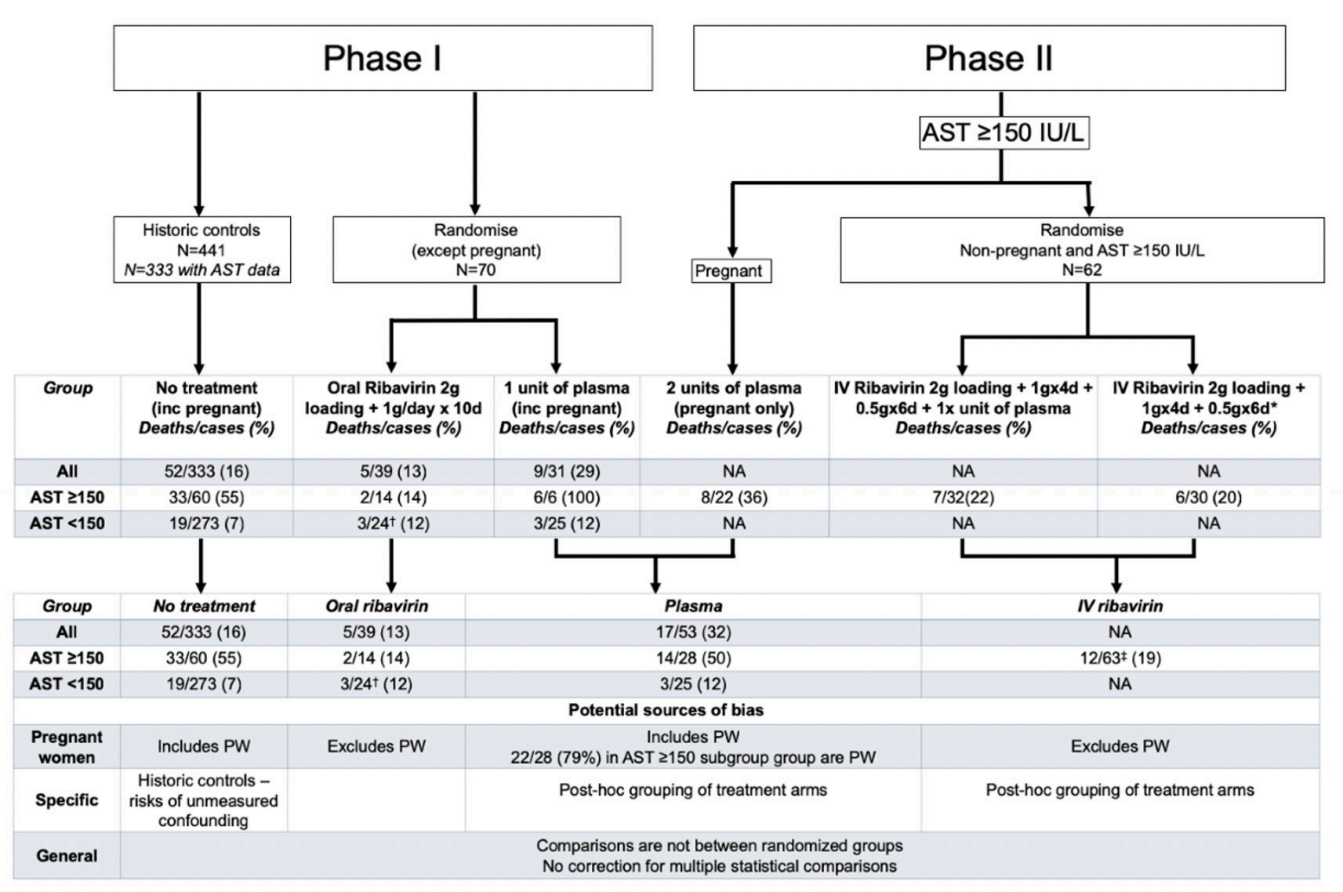
Reconstruction of the McCormick et al. data. AST, aspartate aminotransferase; PW, pregnant women. † Discrepancy within McCormick et al, with 39 patients reported treated with oral ribavirin but only 38 (14+24) outcomes reported. ‡ Discrepancy within McCormick et al, with table 1 reporting 12/63 but text reporting 13/62.

It has been well known among Lassa specialists that the McCormick study reports a subset of a much larger dataset assembled by the Lassa treatment unit in Sierra Leone and that a report on the full dataset was commissioned by the United States Army Medical Research and Development Command. One of us (PH) therefore submitted a freedom of information (FOI) request to access this report. The full report and an accompanying memo are available, and we encourage readers to access and read the materials [7,8]. The memo states that some of the original trial records were unavailable, and the data should be “interpreted with extreme caution.” Nonetheless, the report presents data from 1977 through to 1991 on 807 Lassa fever patients with a known outcome that were assigned to different ribavirin treatment regimens. These newly available data raise important questions about the safety and efficacy of ribavirin for the treatment of Lassa fever.

The original data were lost during the civil war in Sierra Leone, but the report contains tables showing the distribution of characteristics of the whole population according to treatment group, an appendix showing individual data for the 405 patients who died, and results of a logistic regression analysis comparing the effect of ribavirin with no treatment for some of the ribavirin regimens, after adjusting for patient characteristics. Based on these data, we derived aggregated datasets containing the number of deaths according to treatment groups and individual characteristics. We combined groups I (“No treatment given”) and X (“Drugs were not available”) as no treatment and all groups in which ribavirin was administered (II, III, and V to IX) as ribavirin. Exhibit III-8 in the FOI report presented case fatality by treatment group and AST, from which we derived crude odds ratios (ORs) comparing ribavirin with no treatment. The logistic regression reported in Exhibit III-9 was restricted to “those treatment groups that yielded the lowest case fatality rates with respect to untreated patients in the high severity patient illness category” (groups II, III, V, and VII). It was adjusted for age, gender, time to admission, time to treatment, length of stay, and log(AST). We also reconstructed analyses by digitizing the data on individuals who died in Appendix D, calculating the number of deaths according to treatment group and AST, and subtracting these numbers from the totals presented in Exhibit III-2. These allowed us to estimate overall mortality ORs before and after adjusting for ribavirin, although the numbers did not entirely match, and so the number of deaths was reduced in some small groups.

Estimates of the effect of oral and intravenous ribavirin from the McCormick study and of all ribavirin from the full report are shown in Fig 2. Based on the crude ORs derived from Exhibit III-8, ribavirin reduced mortality only in patients with serum AST ≥150 IU/L, with less benefit (OR 0.48 [95% CI 0.30 to 0.78]) than reported by McCormick and colleagues. However, ribavirin appeared to increase mortality in patients with serum AST <150 IU/L (2.90 [1.42 to 5.95]). In fact, in our analysis, the only stratum in which ribavirin appeared protective (0.38 [0.21 to 0.70]) was serum AST >300 IU/L (Table H in S1 Text). The logistic regression reported in the FOI report suggested a modest reduction in mortality, but the reasons for the choice of treatment groups compared were unclear. In the reconstructed analyses, ribavirin was associated with overall increased mortality (2.12 [1.67, 2.68]), although this was attenuated after adjustment for AST (1.48 [1.05, 2.08]).

**Fig 2:**
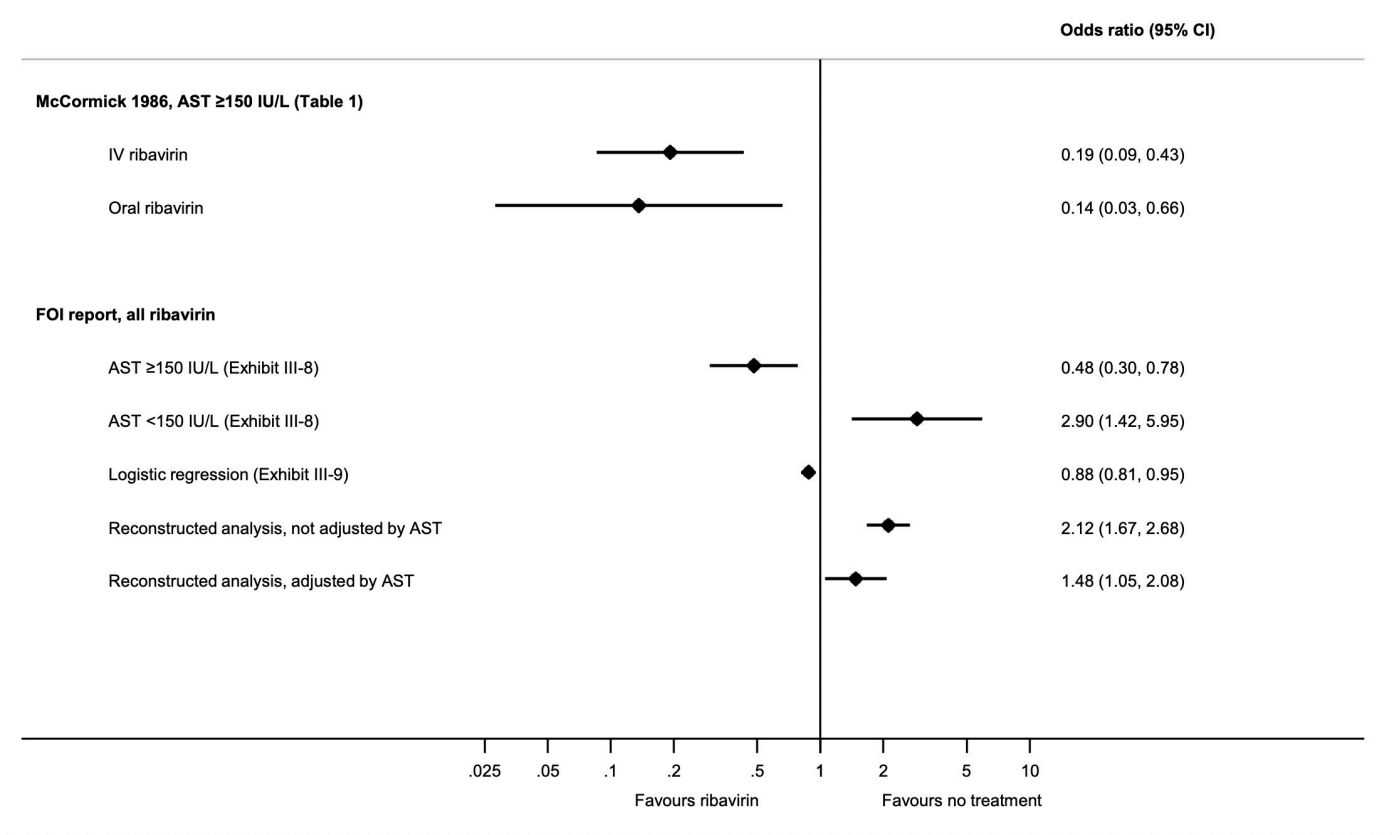
Forest plot of the OR of death in treatment and risk subgroups. AST, aspartate aminotransferase; FOI, freedom of information; OR, odds ratio.

In our view, there is a compelling case to reevaluate the role of ribavirin in the care of patients with Lassa fever. The data suggest that ribavirin treatment may harm Lassa fever patients with AST <150 IU/L. The limitations revealed by the US Army report, such as large amounts of missing data, unclear treatment allocation practices, imbalances in treatment groups, and errors in coding serology results, cast further doubt on the conclusions of the McCormick study. This aligns with 2 recent systematic reviews by Eberhardt and colleagues and Cheng and colleagues, which concluded that the efficacy of ribavirin in Lassa fever was uncertain because of critical risk of bias in existing studies [9,10].

Challenging a quarter of century of clinical practice is difficult. The first step is to acknowledge inadequacies in our knowledge and to ensure that treatment recommendations for Lassa fever better reflect the (weak) strength of evidence for ribavirin in different patient populations. Vigorous efforts should be made to engage clinicians and patients in designing a placebo-controlled trial to assess the safety and efficacy of ribavirin treatment in Lassa fever patients, particularly in those with milder disease (as may be indicated by an admission AST <150 IU/L) in whom the available evidence is compatible with ribavirin causing more harm than good.

In conclusion, Lassa fever patients are receiving a drug that may lack efficacy or cause harm. It is incumbent on us to ensure that the next 25 years of Lassa fever treatment are built on more solid foundations.

## Supporting information

S1 Text Supporting information. (DOCX)
