# Supplementary material for: Time to reconsider the role of ribavirin in Lassa fever
Source: PLoS Negl Trop Dis. 2021 Jul 8;15(7):e0009522. doi: 10.1371/journal.pntd.0009522 (PMC8266111; doi:10.1371/journal.pntd.0009522)
Supplement: S1 Text — (DOCX) [file pntd.0009522.s001.docx]

Time to reconsider the role of ribavirin in Lassa fever: Online supplementary material 1

# Derivation of results from the study by McCormick et al.

Patient characteristics and interventions used in the two phases of the study are summarised in Table A. During Phase I the authors conducted a case-control study among untreated patients and a randomised controlled trial comparing oral ribavirin with Lassa-convalescent plasma. Participants in the case-control study were considered as historical controls in the comparisons presented by McCormick et al [1]. During Phase II, the authors randomly assigned patients with AST ≥150 IU/L to IV ribavirin with or without convalescent plasma. We reconstructed an outcome table from the data reported in Tables 1, 2 and 5 of McCormick et al, together with information in the text of the paper (Table B: footnotes to this table explain details of how data were derived and note discrepancies between numbers reported in the text and tables).

Table A. Summary of two phases in the report (page 21, McCormick et al)

|  | **Patient characteristics** | **Interventions** | **Note** |
| --- | --- | --- | --- |
| *Phase I* | Confirmed Lassa fever cases recruited from a case-control study and a study of oral ribavirin and Lassa-convalescent plasma | Case-control study:   1. No therapy (untreated patient from the case-control study; N = 441)   Randomised study:   1. Oral ribavirin (2g loading dose and 1g in divided eight hours for 10 days (N = 39)) 2. Lassa-convalescent plasma (~ 4mL/kg; 1 unit) with an immunofluorescent-antibody titre ≥ 1:128 within 24 hours of admission (N = 31)) | Pregnant patients were not included in the study and treated with plasma only |
| *Phase II* | Confirmed Lassa fever cases with AST ≥150 IU/L | Patients were randomly assigned to:   1. IV ribavirin (2g loading dose and 1g every 6 hours for 4 days. Then reduced to 0.5g every 8 hours for another 6 days (N = 29)) 2. IV ribavirin (same regimen as 1) + convalescent plasma (1 unit; 300 ml) (N = 33) | Pregnant patients with AST ≥ 150 IU/L were not included in the study and treated with plasma (2 units) only. |

Table B. Summary table of outcomes reported in overall and different sub-groups

|  | **No ribavirin**  (N = 441)^♯^ | **Oral ribavirin**  (N = 39)^¥^ | **IV ribavirin**  (N = 62) | **Plasma**  (N = 53)^₸^ |
| --- | --- | --- | --- | --- |
| *Overall* | | | | |
| Died | 73 | 5 | 13 | 17 |
| Survived | 368 | 33 | 49 | 36 |
| Total | 441 | 38 | 62 | 53 |
| *AST ≥ 150 IU/L (derived from Table 1 in McCormick et al)* | | | | |
| Died | 33 | 2^‡^ | 12^†^ | 14 |
| Survived | 27 | 12^‡^ | 51^†^ | 14 |
| Total | 60 | 14^‡^ | 63^†^ | 28 |
| *AST < 150 IU/L* | | | | |
| Died | 19^*^ | 3^⊗^ | Not applicable | 3^₸^ |
| Survived | 254^*^ | 21^⊗^ | Not applicable | 22^₸^ |
| Total | 273^*^ | 24^⊗^ | Not applicable | 25^₸^ |
| *Viremia ≥ 10^3.6^ TCID_50_/mL (derived from Table 2 in McCormick et al)* | | | | |
| Died | 35 | 3 | 10 | 12 |
| Survived | 11 | 7 | 21 | 9 |
| Total | 46 | 10 | 31 | 21 |
| *Viremia < 10^3.6^ TCID_50_/mL (derived from Table 5 in McCormick et al)* | | | | |
| Died | 31 | 2 | 3 | 5 |
| Survived | 80 | 27 | 29 | 27 |
| Total | 111 | 29 | 31 | 32 |

^♯^ Although there were 441 untreated patients in the case-control study, only 333 of these patients had measured admission levels of AST and 157 had measured serum levels of Lassa virus (page 22, McCormick et al).

* The numbers were derived by subtracting the numbers with AST ≥150 IU/L who died and survived (reported in Table 1) from the total number of patients with measured AST reported in the text on page 22 (McCormick et al): *“We measured the admission levels of AST in 333 untreated patients, of whom 52 (15.6 percent) died.”*.

^†^ The numbers analysed are based on Table 1, which reports 12 deaths among 63 patients with AST ≥150 IU/L treated with IV ribavirin. This is consistent with the results split according to time of treatment reported in in Table 3. There is a slight inconsistency with the numbers reported in the text description on page 23 (13 deaths among 62 patients): *“We observed case-fatality rates of 20 percent (6 of 30) in patients treated with intravenous ribavirin and 22 percent (7 of 32) in patients treated with ribavirin and plasma together. Since these differences were also not significant, and we saw no therapeutic effect from plasma alone, we combined these patients into a single group treated with intravenous ribavirin (62 patients).*”

^₸^ These numbers were derived by subtracting the number of patients and deaths in patients with AST ≥150 IU/L treated with plasma, reported in Table 1, from the total patients treated with plasma (and deaths in these patients) based on the text on page 23: *“We observed a case-fatality rate of 29 percent (9 of 31) in patients treated with 1 unit of Lassa-convalescent plasma; this rate did not differ significantly from the rate in patient treated with 2 units of plasma (36 percent, 8 of 22). Hence, we combined both these patient groups for analysis as the plasma-treated group (53 patients).”*

^‡^ The numbers analysed are based on Table 1, which reports 2 deaths among 14 patients with AST ≥150 IU/L treated with oral ribavirin.

^⊗^ The paper did not report a table for AST <150 IU/L. These numbers were derived from page 23: *“Patients with an admission AST < 150 IU per liter who were treated with oral ribavirin had a case-fatality rate of 12 percent (3 of 24).”* However, the total number of patients with AST ≥150 IU/L treated with oral or IV ribavirin

^¥^ Note that there is an inconsistency in the reported number of patients treated with oral ribavirin. Adding the total numbers of patients with AST <150 IU/L and AST ≥ 150 IU/L (24+14=38, calculated as described above) is inconsistent with the total of 39 reported in the Methods section on page 21: “*One group (39 patients) received oral ribavirin in a 2-g loading dose followed by 1 g per day given in divided doses every eight hours for 10 days”*.

# Derivation of results from IND 16666 report

The treatment groups in IND 16666 report [2] were:

(I) No treatment;

(II) IV Ribavirin followed by oral dose;

(III) Ribavirin + plasma;

(IV) Plasma alone;

(V) Ribavirin 25-30mg loading dose;

(VI) Ribavirin 34mg loading dose;

(VII) Ribavirin 33mg loading dose followed by ¼ dose;

(VIII) Ribavirin 17mg loading dose followed by ⅛ dose;

(IX) Ribavirin + prostacyclin;

(X) Patients for whom no drugs were available.

Data on survival according to treatment group were reported in Exhibit III-2 (‘Percentage Distribution of Patients by Selected Characteristics and by Treatment Group’) and Exhibit III-7 (‘Survivorship Among Treatment Groups’). Exhibit III-2 reported the percentage of patients who died and survived in each treatment group, together with the number of patients with unknown outcome, while Exhibit III-7directly reported on the number of patients who died and survived, among those with known outcome. There were discrepancies between the numbers reported in these two tables. For example, Exhibit III-2 reports that of 601 patients in Treatment group II 140 had unknown outcomes, leaving 461 patients with known outcomes. However, Exhibit III-7 reports that there were 594 patients in this group, of whom 119 patients died and 475 survived.

### Overall results (Exhibit III-7)

We elected to use the data in Exhibit III-7 for analyses, because this table directly reported on mortality according to treatment group. Table C shows the data presented in IND 16666 Exhibit III-7. All ribavirin groups (II, III, and V-IX) were combined and compared with control (combined groups I and X).

Table C. Outcome table derived from Exhibit III-7. Ribavirin treatment groups are shaded in grey.

|  | **Treatment group** | | | | | | | | |
| --- | --- | --- | --- | --- | --- | --- | --- | --- | --- |
|  | **Control (groups 1 and X)** | **II** | **III** | **IV** | **V** | **VI** | **VII** | **VIII** | **IX** |
| **Died** | 145 | 119 | 8 | 19 | 5 | 5 | 4 | 4 | 14 |
| **Survived** | 846 | 475 | 29 | 57 | 29 | 7 | 21 | 5 | 3 |
| **Total** | 991 | 594 | 37 | 76 | 34 | 12 | 25 | 9 | 17 |

### Subgroup results by admission AST levels (Exhibit III-8)

Outcomes according to admission AST status (AST ≥150 IU/L and AST <150 IU/L) were derived from Exhibit III-8 and are presented in Tables D and E. All ribavirin groups (II, III, and V-IX) were combined and compared with control (combined groups I and X). in the forest plot (Figure 1).

Table D. Outcome table derived from Exhibit III-8 (AST ≥ 150 IU/L). Ribavirin treatment groups are shaded in grey.

|  | **Treatment group** | | | | | | | | | |
| --- | --- | --- | --- | --- | --- | --- | --- | --- | --- | --- |
|  | **I** | **II** | **III** | **IV** | **V** | **VI** | **VII** | **VIII** | **IX** | **X** |
| **N** | 56 | 388 | 31 | 25 | 21 | 7 | 20 | 9 | 12 | 26 |
| **%Died** | 44.6 | 25.0 | 22.6 | 44.0 | 19.0 | 57.1 | 20.0 | 44.4 | 75.0 | 38.5 |
| **Died** | 25 | 97 | 7 | 11 | 4 | 4 | 4 | 4 | 9 | 10 |
| **Survived** | 31 | 291 | 24 | 14 | 17 | 3 | 16 | 5 | 3 | 16 |

Table E. Outcome table derived from Exhibit III-8 (AST < 150 IU/L). Ribavirin treatment groups are shaded in grey.

|  | **Treatment group** | | | | | | | | | |
| --- | --- | --- | --- | --- | --- | --- | --- | --- | --- | --- |
|  | **I** | **II** | **III** | **IV** | **V** | **VI** | **VII** | **VIII** | **IX** | **X** |
| **N** | 296 | 154 | 4 | 26 | 10 | 5 | 5 | 0 | 5 | 8 |
| **%Died** | 4.1 | 9.1 | 25.0 | 15.4 | 0 | 20.0 | 0 | 0 | 100.0 | 12.5 |
| **Died** | 12 | 14 | 1 | 4 | 0 | 1 | 0 | 0 | 5 | 1 |
| **Survived** | 284 | 140 | 3 | 22 | 10 | 4 | 5 | 0 | 0 | 7 |

### Overall results adjusted by log(AST) (Exhibit III-9)

The logistic regression results were derived from Exhibit III-9, which combined Treatment groups II, III, V and VII (ribavirin) and Treatment groups I and X (no ribavirin) in the analysis. The IND 1666 authors reported that they selected these treatment groups because they “yielded the lowest case fatality rate with respect to untreated patients in the high severity patient illness category”. The effect of ribavirin was adjusted for gender, interval onset to admission, interval admission to treatment, length of stay and AST (‘log(SGOT)’). The logistic regression coefficient (log odds ratio) was reported as 0.1289, with the outcome coded as died=0, survived=1. The odds ratio was therefore derived as exp(-0.1289) = 0.879. The associated p-value was reported as 0.0015, which corresponds (for a two-tailed test) to a z statistic of 3.175. The standard error of the log odds ratio was derived as coefficient/z = 0406. The coefficient and standard error were used to derive a confidence interval for the log odds ratio, which was converted to a confidence interval for the odds ratio (0.812-0.952). Note that this confidence interval appears too narrow, compared with that for the crude odds ratio.

Table F summarises the results derived from McCormick et al and IND 16666 and presented in Tables B to E, which are displayed in the forest plot (Figure 1).

Table F. Summary of case fatality rates and odds ratios from McCormick et al and IND 16666

|  | Deaths/total (% deaths) | | Odds ratio (95% CI) |
| --- | --- | --- | --- |
|  | **Ribavirin** | **No ribavirin** |  |
| McCormick et al | | | |
| Overall | 18/100 (18.0%) | 73/441 (16.6%) | 1.11 (0.63, 1.95) |
| AST ≥ 150 IU/L | 14/77 (18.1%) | 33/60 (55.0%) | 0.18 (0.08, 0.39) |
| AST < 150 IU/L | 3/24 (12.5%) | 19/273 (7.0%) | 1.91 (0.52, 6.98) |
| IND 16666 | | | |
| Overall (Exhibit III-7) | 159/728 (21.8%) | 145/991 (14.6%) | 1.63 (1.27, 2.09) |
| AST ≥ 150 IU/L (Exhibit III-8) | 129/488 (26.4%) | 35/82 (42.7%) | 0.48 (0.30, 0.78) |
| AST < 150 IU/L (Exhibit III-8) | 21/183 (11.5%) | 13/304 (4.3%) | 2.90 (1.42, 5.95) |
| Overall, adjusted (Exhibit III-9) | - | - | 0.88 (0.81, 0.95) |

**Reconstruction of numbers of individuals and deaths according to admission AST.**

Table G shows the number of individuals according to treatment group and admission AST. These were derived from the numbers and percentages presented in Exhibit III-2 of the report. Table H shows the total number of deaths according to treatment group and admission AST. These were derived from the individual-level data on patients who died presented in Appendix D of the report. In four groups, shown in the Table H, the number of deaths exceeded the number of individuals in the corresponding group. The number of deaths in these four groups was reduced to the number of individuals in Table G.

Table I shows mortality odds ratios for ribavirin compared with no treatment, according to admission AST. These were derived from the data in Tables G and H. The unadjusted and adjusted odds ratios for ribavirin compared with no treatment, shown in Figure 2, were also derived from the data in Tables G and H.

Table G. Total patients according to treatment group and admission AST, reconstructed from Exhibit III-2

|  | Treatment group | | | | | | | | | | |  |
| --- | --- | --- | --- | --- | --- | --- | --- | --- | --- | --- | --- | --- |
| Admission AST | I | II | III | IV | V | VI | VII | VIII | IX | X | Unknown | All patients |
| <150 | 301 | 156 | 4 | 26 | 10 | 5 | 6 | 0 | 5 | 8 | 20 | 540 |
| 150-199 | 8 | 86 | 2 | 5 | 4 | 1 | 7 | 5 | 0 | 3 | 1 | 122 |
| 200-249 | 7 | 54 | 4 | 0 | 7 | 2 | 2 | 1 | 1 | 2 | 4 | 84 |
| 250-299 | 4 | 31 | 1 | 4 | 0 | 0 | 1 | 0 | 0 | 1 | 1 | 43 |
| >300 | 37 | 221 | 24 | 16 | 10 | 4 | 10 | 3 | 11 | 20 | 8 | 365 |
| Unknown | 610 | 53 | 2 | 25 | 3 | 0 | 0 | 0 | 0 | 1 | 2 | 696 |
| Total | 967 | 601 | 37 | 76 | 34 | 12 | 26 | 9 | 17 | 35 | 36 | 1850 |

Table H. Total number of deaths according to treatment group and admission AST, reconstructed from Appendix D

|  | Treatment group | | | | | | | | | |  |
| --- | --- | --- | --- | --- | --- | --- | --- | --- | --- | --- | --- |
| Admission AST | I | II | III | IV | V | VI | VII | VIII | IX | X | Total deaths |
| <150 | 13 | 21 | 1 | 4 | 3 | 2 | 1 | 0 | 5 | 2 | 52 |
| 150-199 | 1 | 11 | 0 | 0 | 1 | 0 | 0 | 1 | 0 | 0 | 14 |
| 200-249 | 1 | 7 | 1 | 0 | 1 | 1 | 0 | 0 | 1 | 0 | 12 |
| 250-299 | 0 | 9 | 0 | 1 | 0 | 0 | 1 | 0 | 0 | 2* | 13 |
| >300 | 23 | 82 | 6 | 10 | 3 | 4 | 13* | 7* | 9 | 14 | 171 |
| Unknown | 97 | 16 | 0 | 4 | 2 | 2* | 0 | 0 | 0 | 0 | 121 |
| Total | 135 | 146 | 8 | 19 | 10 | 9 | 15 | 8 | 15 | 18 | 383 |

* Number of deaths exceeds total number of patients in Table G. In analyses the number of deaths was reduced to the number of individuals in Table G.

Table I. Mortality odds ratios for ribavirin compared with no treatment, according to admission AST.

| Admission AST | Odds ratio (95% CI) |
| --- | --- |
| <150 | 4.23 (2.19, 8.15) |
| 150-199 | 1.41 (0.17, 12.1) |
| 200-249 | 1.47 (0.16, 13.1) |
| 250-299 | 1.74 (0.17, 18.3) |
| >300 | 0.38 (0.21, 0.70) |
| Unknown | 2.38 (1.31, 4.35) |

**References**

1. McCormick JB, King IJ, Webb PA, Scribner CL, Craven RB, Johnson KM, et al. Lassa fever. Effective therapy with ribavirin. N Engl J Med. 1986;314(1):20-6.

2. Birch & Davis Associates and Sherikon Inc. on behalf of the U.S. Army Medical Research and Development Command. Final Report Analysis of a Clinical Trial Ribavirin and the Treatment of Lassa Fever. Maryland, USA. 1992 February. Report No.: IND 16666. Available at: <https://media.tghn.org/medialibrary/2019/03/Responsive_Documents_of_Peter_Horby.pdf.pdf> [Accessed on 19 Apr 2021]. 1992.
